# Application of Bacteriophages to Limit *Campylobacter* in Poultry Production

**DOI:** 10.3389/fmicb.2021.458721

**Published:** 2022-01-05

**Authors:** Elena G. Olson, Andrew C. Micciche, Michael J. Rothrock, Yichao Yang, Steven C. Ricke

**Affiliations:** ^1^Meat Science and Animal Biologics Discovery Program, Department of Animal and Dairy Sciences, University of Wisconsin–Madison, Madison, WI, United States; ^2^Center for Food Safety, Department of Food Science, University of Arkansas, Fayetteville, AR, United States; ^3^Agricultural Research Service, United States Department of Agriculture, Athens, GA, United States; ^4^Department of Poultry Science, University of Arkansas, Fayetteville, AR, United States

**Keywords:** *Campylobacter*, poultry, bacteriophage, post-harvest, pre-harvest

## Abstract

*Campylobacter* is a major foodborne pathogen with over a million United States cases a year and is typically acquired through the consumption of poultry products. The common occurrence of *Campylobacter* as a member of the poultry gastrointestinal tract microbial community remains a challenge for optimizing intervention strategies. Simultaneously, increasing demand for antibiotic-free products has led to the development of several alternative control measures both at the farm and in processing operations. Bacteriophages administered to reduce foodborne pathogens are one of the alternatives that have received renewed interest. *Campylobacter* phages have been isolated from both conventionally and organically raised poultry. Isolated and cultivated *Campylobacter* bacteriophages have been used as an intervention in live birds to target colonized *Campylobacter* in the gastrointestinal tract. Application of *Campylobacter* phages to poultry carcasses has also been explored as a strategy to reduce *Campylobacter* levels during poultry processing. This review will focus on the biology and ecology of *Campylobacter* bacteriophages in poultry production followed by discussion on current and potential applications as an intervention strategy to reduce *Campylobacter* occurrence in poultry production.

## Introduction

According to the World Health Organization, *Campylobacter* is a leading cause of the diarrheal disease (World Health Organization [WHO], 2018). The genus *Campylobacter* is comprised of over 20 species. Strains of *Campylobacter jejuni* and *Campylobacter coli* are generally considered some of the more significant concerns among foodborne pathogens for human health (Korczak et al., 2006; Havelaar et al., 2012; World Health Organization [WHO], 2018; Centers for Disease Control and Prevention [CDC] (2019)). European Food Safety Authority (EFSA) has declared campylobacteriosis as being one of the most commonly reported foodborne diseases since 2005, with over 200,000 cases per year, representing 70% of the human zoonoses in the E.U. (European Food Safety Authority [EFSA], 2020). In one study in the United Kingdom (U.K.), it was reported that 50–80% of poultry harbored *Campylobacter* in their intestinal tract (Connerton et al., 2011; European Food Safety Authority [EFSA], 2011, 2020). These numbers are similar to those found in poultry produced in the United States (U.S.) (Hanning et al., 2010; Chapman et al., 2016).

With populations in the bird cecum in some instances exceeding seven log_10_ colony-forming units (CFU) per gram of cecal content, this genus appears to be specifically well suited to reside within the poultry gastrointestinal tract (GIT) (Rudi et al., 2004; Connerton et al., 2011; Indikova et al., 2015). Consequently, *Campylobacter* can be released from the poultry GIT as birds are being processed, potentially contaminating poultry processing plant equipment and the finished product (Elvers et al., 2011; García-Sánchez et al., 2017). Furthermore, *Campylobacter* contamination in the poultry processing plant can remain a persistent problem. Given the poultry GIT establishment and subsequent likelihood of contamination in the poultry plant, poultry meat products are considered a significant source of potential infection for human campylobacteriosis (Umaraw et al., 2017). Pre-and postharvest interventions have been utilized and proposed over the years to reduce *Campylobacter* populations in poultry (Umaraw et al., 2017; Kim et al., 2019; Deng et al., 2020). One intervention that has received more interest as a potential intervention for *Campylobacter* is the administration of *Campylobacter* phages. This review aims to focus specifically on the ecology of *Campylobacter* phages, their mechanisms of bacterial host infection, host resistance, and their applications in both pre-and postharvest as intervention strategies toward reducing *Campylobacter* in poultry production. Due to the lytic characteristic that phages possess, multi-pronged interventions that include combination of phages with non-phage technologies, such as acids and endolysins, throughout poultry processing steps may result in efficient reduction of *Campylobacter* under commercial conditions.

## *Campylobacter* Phage – Classification and Mechanisms of Host Infection

The first *Campylobacter* phages were likely isolated in 1960 in cattle and pigs from then identified *Vibrio coli* and *Vibrio fetus*, now known as *C. coli* and *C. fetu*s (Fletcher and Bertschinger, 1964; Fletcher, 1968). A few of the over 170 known phages infect *C. jejuni*, and most of them are specific to the bacterial host (Ushanov et al., 2020). Almost all currently isolated phages that infect *Campylobacter* are from the family *Myoviridae* and morphologically distinguished as Bradley’s morphotype A1 with a contractile tail (Gencay et al., 2018; Jäckel et al., 2019; Ushanov et al., 2020; Table 1). Some *Campylobacter* bacteriophages are from the family of *Siphoviridae* and possess Bradley’s morphotype B1 with a non-contractile tail (Ushanov et al., 2020). Furthermore, lytic phages that target *Campylobacter* are typically assembled into three groupings (I, II, III) based on genome size (Jäckel et al., 2019; Ushanov et al., 2020). Group I lytic phages are 320 kb in genome size and considered unstable, with only two isolates being identified (Frost et al., 1999; Connerton et al., 2018). Although both group II and III have been demonstrated to be useful for phage therapy, most isolated *Campylobacter* phages belong to group III (Sails et al., 1998; El-Shibiny et al., 2009; Carvalho et al., 2010). Group II phages with an average genome size of 162,601 base pairs, and Group III phages (133, 166 base pairs) comprise approximately half the group I phages’ genomic size (Jäckel et al., 2019). Group II phages have a protein head diameter of 83 to 99 nm (Carvalho et al., 2010; Jäckel et al., 2019). Group III phages have a head diameter of 100–130 nm, whereas Group I phages possess much larger head proportions (Jäckel et al., 2019). Based on whole genome sequencing and protein analysis, groups II and III bacteriophages can be further combined into the Eucampyvirinae sub-family (Ushanov et al., 2020). Zampara et al. (2017) further grouped *C. jejuni* phages based on receptor dependency, namely, group III phages use CPS receptors, and group II phages contact the host *via* flagella.

**TABLE 1:** *Campylobacter* phages taxonomy and description.

Order (Harper et al., 2014)	Family (Sharp, 2001; Gencay et al., 2018; Huang et al., 2020; Ushanov et al., 2020)	Nucleic acid type and phage example (Harada et al., 2018)	Morphotype (Sails et al., 1998)	Grouping not based on genome analysis (Jäckel et al., 2019)	Genome size (Huang et al., 2020)	Applicability (Carvalho et al., 2010; Connerton et al., 2018; Huang et al., 2020)	Genera based on genome sequencing (Javed et al., 2014)	Phages (Javed et al., 2014; Jäckel et al., 2019; Huang et al., 2020)	Phage resistance development (Janez and Loc-Carillo, 2013)	Host range (Jäckel et al., 2019; Huang et al., 2020)
Caudovirales	*Myoviridae*	Linear dsDNA (T4)	Morphotype A1 with contractile tail	Group I	∼320 kb	Unstable			Motility defect	
				Group II	Average ∼162,601	Stable for phage therapy	CP220virus	CP21, CP220, Cpt10, vB-Ccom-IBB-35	Motility defect	*C. jejuni* and *C. coli*
				Group III	Average ∼133,166	Stable for phage therapy	CP8virus	CP81, CPX, NCTC12673, Cp30A, PC14, PC5, vB_CjeM_Los1, CP8	CPS structure	*C. jejuni*
	*Siphoviridae*	Linear dsDNA (Lambda)	Morphotype B1 with non-contractile tail		15–17 kb	Stable for phage therapy		CAM-P21		*C. coli*
	*Podoviridae*									

Several phage receptors in Gram-negative bacteria have been recognized, which consist of bacterial surface components such as lipopolysaccharides (LPS), CPS, flagella, outer membrane proteins (Omps), and porins (Rakhuba et al., 2010). For instance, the T-even phages, such as *Escherichia coli* T4 phage, which is the model of the *Myoviridae* phages having contractile tail structure, are among the best-characterized phages (Sørensen et al., 2011). T-even phages recognize and bind to a variety of Omps or specific structures within LPS in *E. coli*. Bacterial receptors in other Gram-negative bacteria that are recognized by phages consist of structures within LPS such as O antigens and carbohydrate moieties (Kiljunen et al., 2005; Petty et al., 2007). The O antigens are central features of *E. coli* and *C. jejuni* cells surface and represent essential factors of infection and disease associated with humans (Mills et al., 1992; Stenutz et al., 2006). Application of phages that bind O antigens may help reduce the virulence of pathogens such as *Campylobacter* within human GIT and thus may be beneficial in postharvest applications for poultry meat intended for retail destinations such as ready to eat meats.

## *Campylobacter* Phage-Ecology

*Campylobacter* phages have been isolated wherever their hosts exist, such as the feces of sheep, cows, pigs (Hansen et al., 2007; Rizzo et al., 2015; An et al., 2018); slaughterhouse run-offs, sewage, manure, excreta of chickens and their meat (Grajewski et al., 1985; Salama et al., 1989; Sails et al., 1998; Atterbury et al., 2005; Connerton et al., 2004, 2011; El-Shibiny et al., 2005; Loc Carrillo et al., 2007; Tsuei et al., 2007). Reports on the isolation of bacteriophages from poultry are considerably variable. For example, out of 205 broiler ceca, approximately 20% were positive for *Campylobacter* bacteriophages in the U.K., and a similar result was observed with broilers in South Korea (Atterbury et al., 2005; Hwang et al., 2009). However, in a Denmark study, the *Campylobacter* bacteriophages’ isolation rate from conventionally raised broiler intestines was only 3% (Hansen et al., 2007). In contrast to these low rates of isolation, Owens et al. (2013) reported that 100% of the fecal samples from free-range broilers and egg layers tested positive for *C. jejuni* phages. El-Shibiny et al. (2005) isolated 51% *Campylobacter* phages from the *Campylobacter*-positive organic chickens from a U.K. flock. This increase makes sense because free-range birds potentially encounter a wider variety of *Campylobacter* species and their phages because they are exposed to a broader range of environmental surroundings (Hald et al., 2001; Atterbury et al., 2003b). In general, the likelihood of phage recovery potentially increases with the presence of a susceptible host; therefore, birds with higher colonization rates of *Campylobacter* are more likely to be sources of phages (Atterbury et al., 2003b).

*Campylobacter* colonization in poultry can vary considerably and this can impact the contamination levels throughout the poultry production chain. In a study by Rudi et al. (2004), the concentration of *C. jejuni* in poultry ceca exhibited a 1,000-fold difference in range throughout a number of flocks. If the strain colonizing one community has an infectious dose that is 0.1% of the strain colonizing another flock, then these two strains would be considered relatively equivalent in their likelihood to cause illness through food contamination (from a human infectivity standpoint) (Rudi et al., 2004). Since up to 76% of chickens slaughtered can be *Campylobacter* positive, contamination management continues to be an important challenge to poultry production (Humphrey et al., 1993). In addition, with cross-contamination occurring in the slaughterhouse, *Campylobacter* and phages discovered on a single chicken carcass may have originated from more than one source (Atterbury et al., 2003b). Although, tracking of phages might be difficult throughout poultry processing due to cross-contamination effects, the capability of phages to persist throughout poultry processing demonstrated by Atterbury et al. (2003a) is an essential characteristic of their future use in the biocontrol of *Campylobacter* in poultry processing. The phages isolated in this study exhibited a broad range of recovery rates from chicken skin stored at 4°C (Atterbury et al., 2003a). Furthermore, the stated detection limit for phage recovery of 2 × 10^3^ PFU/10 cm^2^ of chicken skin suggests that there are at least that many phages persisting throughout the commercial poultry processing and packaging operation.

## Isolation, Propagation, Concentration, and Purification of *Campylobacter* Phages

**FIGURE 1:**
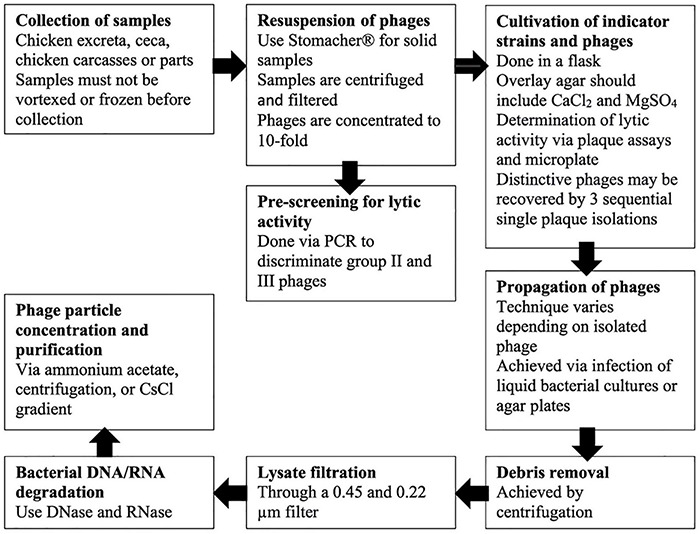
Outline of main concepts of isolation, propagation, concentration, and purification of *Campylobacter* phages.

Isolation strategies may impact both the extent and the type of *Campylobacter* phages detected in different ecosystems. The outline and main concepts for the following procedures are briefly described in Figure 1. The first step for isolation is collecting the samples. Samples should not be frozen or vortexed as these treatments may significantly reduce the plaque-forming ability (Atterbury et al., 2003a; Jäckel et al., 2017). Although Atterbury et al. (2003a) were able to isolate *Campylobacter* phages from over 10% of chilled chicken thighs by plaque assay, Jäckel et al. (2017) reported reduced lytic activity of phages from samples that were previously frozen.

Even though phages have been detected with polymerase chain reaction (PCR) assays in frozen meats, they either did not exhibit lytic activity, were apparently unstable, or simply more difficult to propagate (Janež et al., 2014; Jäckel et al., 2017). Solid samples can be incubated in sodium chloride/magnesium sulfate (S.M.) buffer to resuspend the phages, and the use of a homogenizer, such as a Stomacher^®^, to remove phages from chicken skin provides the best results (Jäckel et al., 2019). Following the centrifugation of the resuspended samples and consequent filtration (0.45 and 0.22 μm) of the supernatant, samples can be scanned for lytic activity (Jäckel et al., 2019). The phages are then concentrated to a 10-fold concentration using centrifugal filter units before spotting on indicator strains (Jäckel et al., 2019). Pre-screening to discriminate group II and group III phages rapidly may be beneficial. Using PCR, Jäckel et al. (2017) distinguished over 45% of total phages as group II or III phages. PCR positive samples that do not show lytic activity with the indicator strain can be tested with other potential strains. Likewise, the PCR negative samples should be examined for lytic activity as they may contain rare group I phages (Jäckel et al., 2017).

The choice of a bacterial host is essential while harvesting the phages. *C. jejuni* NCTC12662 (PT14) is generally used as an indicator strain because it is vulnerable to a broad range of phages, although little is known about its response to phage infection (Hansen et al., 2007; Sørensen et al., 2015; Gencay et al., 2018). PT14 was isolated from chicken ceca, and its complete genome has been sequenced (Brathwaite et al., 2013; Sørensen et al., 2015). Nonetheless, a broad range of candidate host strains consisting of several *fla*-types and Penner serotypes should be considered since group II binds to receptors on the flagellum and group III phages binds to CPS receptors (Sails et al., 1998; Hammerl et al., 2011; Sørensen et al., 2011, 2015; Sorensen et al., 2017; Gencay et al., 2018). Sorensen et al. (2017) developed a protocol to determine receptor dependency of *Campylobacter* phages.

Jäckel et al. (2019) suggested that cultivation of indicator strains be *via Campylobacter* media in flasks rather than tubes, as amplification of bacteria is increased by an ample surface headspace for gas exchange. The selection of an overlay agar, such as NZCYM, is critical for the results of the activity tests and should include CaCl_2_ and MgSO_4_, which enable the attachment of phages to their host cell (Sails et al., 1998; Frost et al., 1999; Sambrook and Russell, 2001). Determination of lytic activity can be achieved *via* plaque assays and microplate tests (Fischer et al., 2013). In addition, to obtain single plaques, dilutions of phage preparations must be plated. The plaques formed by lytic *Campylobacter* phages are generally about one millimeter in diameter and somewhat turbid, so a zoom stereo microscope may be helpful to spot and count plaques (Jäckel et al., 2019). Specific phages may then be recovered by three sequential single plaque isolations (Hammerl et al., 2011; Jäckel et al., 2015).

Optimal propagation technique should be determined for each phage as propagation methods are not equally suited for all *Campylobacter* phages (Hammerl et al., 2011; Jäckel et al., 2015; Gencay et al., 2017; Sorensen et al., 2017). Propagation of lytic phages can be accomplished by either infection of bacterial cultures or creating agar plates and demonstrating confluent lysis (Green and Sambrook, 2012). Hammerl et al. (2011, 2014) and Jäckel et al. (2015, 2019) achieved over 10^8^ PFU/mL by infecting 100 mL cultures of the indicator strain with OD588 of approximately 0.4 with phages at a multiplicity of infection (MOI) of 0.01 followed by incubation for 12–24 h at 42°C. The cultures that appear to grow best in a flask with a filtered stopper can be placed in a box with a gas-producing sachet to mimic ideal growth conditions and incubated while mildly shaken (Jäckel et al., 2019). Since it is not recommended to use high centrifuge force, there is a possibility that remnants of agar can remain, which in turn may worsen filtration. This method has the additional benefit that the mass lysate does not include any agar, which may interfere with filtration (Jäckel et al., 2019). However, in another technique utilized by Loc Carrillo et al. (2007), Owens et al. (2013), and Gencay et al. (2017), the overlay agar is not harvested; instead, the visible plaques are extracted and suspended in buffer using the typical steps described by Frost et al. (1999).

Before the lysate is filtrated through a 0.45 and 0.22 μm filter, the residual agar, cells, and debris are removed by centrifugation (Jäckel et al., 2019). Bacterial DNA and RNA are then degraded using 20 mg/mL of DNase, and RNase added to the lysate and incubated at 37°C for 30–60 min (Jäckel et al., 2019). Phage particles can be subsequently concentrated using a variety of techniques depending on the amount of the lysate, such as ammonium acetate purification done by Ackermann (2009), centrifugal force utilized in Loc Carrillo et al. (2007), or CsCl density gradient done by Hammerl et al. (2011) and Jäckel et al. (2015). CsCl density gradient is also used to purify *Campylobacter* phages, where the application of 10^9^ phage particles is suggested to obtain a prominent band (Jäckel et al., 2019). The extracted phages can consequently be applied to various studies such as morphological typing, selection of host range, and genomic or protein analysis.

## Isolation and Analysis of *Campylobacter* Phage DNA

All sequenced *Campylobacter* phages have double-stranded DNA (Hammerl et al., 2011; Jäckel et al., 2015, 2019; Harada et al., 2018). Furthermore, genomic differences between group II and group III phages affect the choice of DNA extraction method (Hammerl et al., 2011; Jäckel et al., 2015, 2019). For instance, although the standard protocol described by Green and Sambrook (2012) is suitable for the extraction of group II DNA, the use of phenol-chloroform fails in the extraction of group III DNA (Hammerl et al., 2011; Jäckel et al., 2015, 2019). Commercially available kits for phage DNA extraction are available for both phage groups.

*Campylobacter* is resistant against digestion by numerous restriction endonucleases, such as *Ava*II, *Bam*HI, CIaI, *Eco*RV, *Eco*RI, *Hae*III, Hinfl, *Hin*dIII, HpaIII, PstL, PvuIl, Rsal, and Scal (Sails et al., 1998). However, PFGE analysis using restriction endonucleases that cut pure A/T sequences, such as Dral, Smil, or Vspl, can be performed to establish the genome size of the phages and to assign them to their respective groups (Loc Carrillo et al., 2007; Hammerl et al., 2011; Sorensen et al., 2017). Restriction patterns can subsequently be analyzed on standard agarose gel yielding a more rapid and cost-effective evaluation. Jäckel et al. (2019) suggest applying DreamTaq DNA polymerase amplification constituents or whole genome amplification kits for phage DNA amplification such as those used by Hammerl et al. (2011). To date, all *Campylobacter* phage genomes have been sequenced by short-read sequencing, which is predisposed to homopolymer errors (Jäckel et al., 2019). Long DNA repeats that occur in group II phages hinder the assembly of reads using short-read sequencing. Long-read sequencing platforms such as PacBio or MinION can resolve the problem but necessitate a high amount of DNA (2–10 g) of phages, which can be challenging to obtain (Jäckel et al., 2019).

## Poultry Preharvest *Campylobacter* Phage Therapy

**FIGURE 2:**
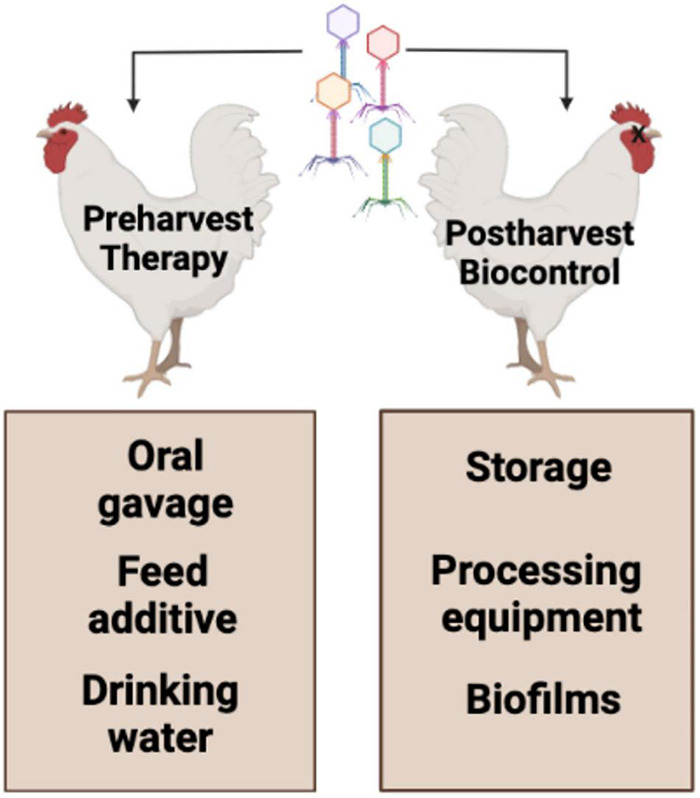
Poultry pre- and post-harvest phage applicability. Figure was created with Biorender.com.

Research is ongoing to reduce pathogen occurrence on poultry farms for *Campylobacter* (Deng et al., 2020). Decreasing pathogen concentrations on poultry farms can affect the reduction of pathogen populations entering the food chain. Preharvest strategies include successful oral application of phages to reduce *C. jejuni* colonization in birds (Figure 2; Carvalho et al., 2010) and phages against *C. jejuni* as an alternative feed additive (Kittler et al., 2013). The phage-bacterial interaction is a typical association in the chicken GIT. Lower levels of *Campylobacter* have been observed to innately occur in the ceca of chickens (5.1 log_10_ CFU/g) in the presence of indigenous *Campylobacter* phages compared to those chickens’ lacking phages in the ceca (6.9 log_10_ CFU/g) (Atterbury et al., 2005). Thus, the majority of preharvest intervention strategies of *Campylobacter* are focused on the reduction or removal of the microorganism from the ceca (Callaway et al., 2004; Hermans et al., 2011; Wheeler et al., 2014; Kim et al., 2019; Deng et al., 2020).

Bacteriophage treatment of *Campylobacter* in chickens was first reported by Loc Carrillo et al. (2005) and Wagenaar et al. (2005). Wagenaar et al. (2005) studied the effects of preventative versus therapeutic phage applications. The study consisted of a 10-day phage treatment trial with the preventive group infected with *C. jejuni* on day four of phage treatment, whereas in the therapeutic group treatment with phage was administered on sixth day of *C. jejuni* infection. Both groups had at least a two log_10_ CFU/g *C. jejuni* reduction, which tapered off within a week to one log_10_ lower than the untreated group. Wagenaar et al. (2005) compared single phage application versus a cocktail of group III phages *via* birds’ oral administration (Table 2). In their study, while the initial reduction was only maintained for 48 h, a one log_10_ CFU/g decrease was sustained after 30 days, independent of when the therapy was administered. In addition, the birds utilized in this study were at the age for slaughter (day 42), indicating that, despite the phage effectiveness peaking at 48 h, *Campylobacter* concentrations could still be reduced in time for processing. More importantly, when Wagenaar et al. (2005) added another group III phage 69 along with phage 71, they observed a 1.5 log_10_ CFU/g reduction that eventually leveled off at one log lower than the untreated birds, which suggested a synergistic effect when phages were applied simultaneously (Wagenaar et al., 2005). Ultimately, Wagenaar et al. (2005) conducted the first *in vivo* study that indicated no signs of pathology to the chickens despite a dose phage administration. Although bacterial and phage strains were not obtained from the poultry meat or excreta, the model is not considered appropriate for broader remedial purposes in poultry manufacturing.

**TABLE 2:** *Campylobacter* phage treatments during *in vivo* preharvest studies.

Phage	Group	Source	Administration	Flock age	*Campylobacter* spp.	Outcome	Developed resistance	Time of sustained reduction	Study
Phage 71 (NCTC 12671)	Group III	NCTC	∼10^10^ PFU by oral gavage	32 days	*C. jejuni* C356	3 log_10_ CFU/g reduction in 24 h in cecal contents	n/a	1 log sustained reduction over 30 days compared to control in both trials	Wagenaar et al., 2005
Phage 71, Phage 69	Group III		∼10^10^ PFU by oral gavage		*C. jejuni* C356	1.5 log_10_ CFU/g initial reduction			
CP8	Group III	Broiler chicken excreta, free-range layer chicken excreta, processed chicken meat	7 log_10_ PFU by oral gavage	25 days	*C. jejuni* GIIC8	5.6 log_10_ CFU/g cecal content	<4%	2.1–1.8 log_10_ sustained reduction for 5 days	Loc Carrillo et al., 2005
CP34	Group III		7 log_10_ PFU by oral gavage		*C. jejuni* HPC5	3.9 log_10_ CFU/g cecal counts			
CP220	Group II	Poultry sources	7 log_10_ PFU by oral gavage	20 days	*C. jejuni* HPC5	2.1 log_10_ CFU/g reduction 24 h post phage	2%	2 log_10_ sustained reduction for 2 days	El-Shibiny et al., 2009
CP220	Group II		9 log_10_ PFU by oral gavage		*C. coli* OR12	2 log_10_ reduction			
PhiCcoIBB35, PhiCcoIBB37, PhiCcoIBB12	Group II	Poultry intestinal contents	1 × 10^6^ PFU phage cocktail by oral gavage or 1.5 × 107 PFU through feed	7 days	*C. jejuni* 2140 CD1 and *C. coli* A11	1.25 log_10_ CFU/g reduction in feces by oral gavage 2 days post administration; 2 log10 CFU/g reduction in feces by feed route 2 days post administration	13%	1.7 log_10_ CFU/g sustained reduction in feces by oral gavage 7 days post administration; 2 log10 CFU/g sustained reduction in feces by feed route 7 days post administration	Carvalho et al., 2010
NCTC 12672, NCTC 12673, NCTC 12674, NCTC 12678	Group III	NCTC	Phage cocktail of 7.4 log_10_ PFU/bird *via* drinking water	36 d	*C. jejuni* NCTC 12661, *C. jejuni* NCTC 12664, *C. jejuni* NCTC 12660	3.2 log CFU/g reduction in cecal content compared to control 1 day post administration	n/a	1.66–2.14 PFU/g sustained reduction in cecal content 6 days post administration	Kittler et al., 2013
NCTC 12673	Group III	NCTC	10^7^ PFU into crop of broiler	9 d	*C. jejuni* 1474-06	1.3 CFU/g log_10_ reduction of cecal contents compared to control up to 3 days post administration	Initially 43%	2.8 log_10_ sustained reduction 21 days post administration in trial II	Fischer et al., 2013
NCTC 12673, NCTC 12674, NCTC 12678, NCTC 12672	Group III	NCTC	Phage cocktail of 10^7^ PFU directly into crop of broiler		*C. jejuni* 1474-06	1.3 CFU/g log_10_ reduction of cecal content compared to control	Initially 24%		
CP14	Group III	Chicken fecal samples of organic origin	5 × 10^8^ PFU by oral gavage	27 days	*C. jejuni* 3871	1 log_10_ reduction of cecal contents	5%	*C. jejuni* counts increased 4 days post administration	Hammerl et al., 2014
CP14, CP81	Group III	Retail chicken	5 × 10^8^ PFU by oral gavage			No reduction	7%(CP14); 8% (CP81)		
CP14, 24 h later CP68	Group II, III	Chicken fecal samples of organic origin	5 × 10^8^ PFU of CP14; 5 × 10^10^ PFU of CP68 by oral gavage			3 log_10_ reduction of cecal contents 2 days post administration of CP68	5% (CP14) 2% (CP68)	*C. jejun*i counts increased 4 days post administration but were still lower than in control group and group with CP14 alone	
CP20, CP30A	Group II, III	Commercial broiler chicken excreta	7 log_10_ PFU *via* oral gavage	24 d	*C. jejuni* HPC5	2.4 log CFU g^–1^ reduction of cecal contents 2 days post treatment.	0.1	1.3 log_10_ CFU g^–1^ sustained reduction after 5 days	Richards et al., 2019

In the same year, Loc Carrillo et al. (2005) demonstrated that phage treatment of birds infected with *C. jejuni* reduced the cecal concentration of the marker strains by 0.5–5 log_10_ CFU/g of cecal contents over 5 days compared to the control birds. The decrease in bacterial load was determined by the phage load, phage-*Campylobacter* grouping, and post-administration time (Loc Carrillo et al., 2005). Loc Carillo and associates used two group III *Campylobacter* phages (CP8 and CP34) in 25-day old broilers administered *via* oral gavage with an antacid (Table 2). Interestingly, phage CP8 was active against one tested strain (*C. jejuni* GIIC8) and not the other (*C. jejuni* HPC5). In contrast, phage CP34 reduced both marker strains and sustained the reduction for 5 days (Loc Carrillo et al., 2005). Host strain specificity has been demonstrated as a potential therapeutic drawback of using *Campylobacter* phages. Few phages infect distinctive bacterial species, and the host range for most of them contains various strains of one bacterial species (Loc Carrillo et al., 2007). Similar to the Loc Carrillo findings, when phage CP8 tested against *C. jejuni* strain HPC5 *in vitro*, no statistically significant reduction was detected by Rabinovitch et al. (2003).

Multiplicity of infection is the ratio of adsorbed or infecting phages to predisposed hosts. Assuming the unlimited time for adsorption, the MOI ratio denotes the threshold of the infecting ratio (Abedon, 2011). Knowing the MOI value is essential for optimizing efficacy. For instance, when the MOI is too high, virions may inactivate through clumping and aggregation (Brown and Bidle, 2014; Roach and Debarbieux, 2017). In addition, MOI depends on the host-phage interaction and varies with medium, phage, and host (Brown and Bidle, 2014; Roach and Debarbieux, 2017). As such, an MOI above the point of saturation of infection, which is host-phage specific, may explain why a higher concentration of phages would not further reduce bacterial concentrations (Kasman et al., 2002; Rabinovitch et al., 2003; Brown and Bidle, 2014). Interestingly, Loc Carrillo et al. (2005) observed that the administration of 9 log_10_ PFU was less effective than 7 log_10_ PFU, which may be explained by potential phage aggregation and non-specific association that can occur once the MOI is achieved. One notable drawback with the Loc Carrillo et al. (2005) and Wagenaar et al. (2005) studies is their phage therapy testing employed only one or two *C. jejuni* strains. While these studies demonstrated reductions in tested strains by phage therapy, they did not evaluate how the phage treatment would interact with a more diverse array of background *Campylobacter* strains.

El-Shibiny et al. (2009) utilized *Campylobacter* group II phage CP220 against *C*. *jejuni* and *C*. *coli* colonized 20-day old broilers and observed a 2-log_10_ CFU/g reduction in cecal *C*. *jejuni* HPC5 populations in 48 h with a single 7-log_10_ PFU dose (Table 2). However, to accomplish a comparable decrease in *C*. *coli* OR12-colonized chickens, a 9-log PFU dose of CP220 was necessary (El-Shibiny et al., 2009). The critical distinction observed between CP220 and group III phages by El-Shibiny et al. (2009) demonstrated the ability of group II phages to lyse *C*. *coli* OR12 and 30% of other broiler strains, including the ones that were not lysed by group III phages. A cocktail mixture of two groups could contribute to a broader host range to give the most coverage of *Campylobacter* species. Physiologically, *C. coli* and *C. jejuni* are similar with comparable cell wall structure and energy metabolism. The primary difference is the ability of *C. jejuni* to hydrolyze hippurate, a compound commonly detected in urine (Totten et al., 1987; Jauk et al., 2003). However, the genomes of *C. jejuni* and *C. coli* are approximately 12% divergent (Sheppard and Maiden, 2015). Thus, it may be possible for phages to infect and lyse *C. coli* but unable to replicate due to variations within the bacterial host cellular machinery. While the phenomenon has not been studied within *Campylobacter* phages, it has been observed that bacterial lysis can occur without phage propagation in the presence of a high MOI environment (Cairns et al., 2009; Abedon, 2011). Therefore, as El-Shibiny et al. (2009) suggested, this may offer a possible explanation for the need of a higher concentration of phages to reduce *C. coli* populations compared to *C. jejuni* populations.

The route of phage administration was examined by Carvalho et al. (2010). In their study the authors evaluated the success of the two different paths for phage application *via* oral gavage or feed intake (Table 2). In the first study, an orally gavaged cocktail of three broad-spectrum phages (phiCcoIBB35, phiCcoIBB37, phiCcoIBB12) was administered to 1-week-old birds infected with *C. jejuni* 2140CD1. In the second study, Carvalho et al. (2010) compared the oral gavage route and application of phages in feed on birds colonized with *C. coli* A11. Administration of phages *via* the feed route resulted in a higher reduction of *Campylobacter* fecal counts versus the oral route 4 days post administration (Table 2; Carvalho et al., 2010). The results of the Carvalho et al. (2010) study indicated that a successful reduction of the two most prevalent *Campylobacter* strains in poultry is possible through the administration of a phage cocktail. In addition, better reduction of *Campylobacter* counts *via* feed intake is advantageous. It is a simple and more practical method of applying phage therapy on farms than oral gavage.

Another route of phage therapy where the cocktail was administered through the drinking water was assessed by Kittler et al. (2013). Despite utilizing the same feed, vaccinations, and living conditions, phages were only significantly effective in reducing *Campylobacter* in one of the three trials, where *Campylobacter* populations were reduced below the detection limit in fecal samples (less than 50 CFU/g) (Kittler et al., 2013). Within 1 day of phage administration *via* drinking water *C. jejuni* cecal counts decreased more than three log_10_ CFU/g in the cecal contents. Three days post administration, at slaughter, *Campylobacter* counts were still reduced by 1.66–2.14 log_10_ CFU/g (Kittler et al., 2013; Table 2). Similar to previously described studies, at least one log_10_ *Campylobacter* reduction could be detected 6 days post-administration, indicating successful self-replication and pathogen biocontrol (Table 2).

Fischer et al. (2013) compared the combinatory effect of a four-phage cocktail to a single phage administration versus *C*. *jejuni* counts over time. Using group III one phage or a four-phage cocktail administered at eight time points over 4 weeks directly into the crop of broilers, *Campylobacter* concentrations within the ceca were monitored over days 1, 3, 7, 14, 21, 28, 35, and 42 after phage application (Fischer et al., 2013). Over time, significant reductions were observed, with the highest decrease being log_10_ 2.8 CFU *Campylobacter*/g of cecal contents on day 21 in both groups (Fischer et al., 2013). When the phage was utilized in a cocktail containing an additional three phages, no significant differences between the cocktail treatment and the single phage treatment were observed (Fischer et al., 2013). The drawback of the study is that Fischer et al. (2013) only used group III phages in a cocktail, which may explain no significant difference when compared to the one phage application. Because the group III phages bind to the same receptors on *Campylobacter* and the phages replicate based on bacterial density, this may explain why the use of group III phages as cocktail and a single phage could produce similar results.

Hammerl et al. (2014) compared combinations of group II (CP68) and III phages with a cocktail of only group III phages (CP14 and CP81) administered *via* oral gavage against *Campylobacter* (Table 2). While a combination of group III phages did not elicit a detectable *C. jejuni* reduction, application of CP14 phage alone achieved one log_10_ CFU/g reduction. These results were similar to the reduction of *Campylobacter* counts achieved by Wagenaar et al. (2005) using only one phage cocktail. However, Hammerl et al. (2014) observed that a sequential combination of CP14 phage followed by CP68 phage led to a three log_10_ CFU/g *Campylobacter* reduction in the cecal contents. Similar to the observations of El-Shibiny et al. (2009), Hammerl et al. (2014) concluded that phage cocktails should be composed of group II and group III phages to successfully combat *Campylobacter.*

Furthermore, Richards et al. (2019) showed that the utilization of phage therapy offers a minimal targeted intervention that is not harmful to the intestinal microbiota of broilers. Richards et al. (2019) demonstrated that a cocktail of CP30A and CP20 phages against *C. jejuni* colonized birds produced significant reductions in intestinal *C. jejuni* populations compared to control birds over 5 days and did not affect the alpha-diversity and richness of microbiota in ceca and ileum of birds compared to control (Table 2). The phage cocktail produced the most *Campylobacter* reduction in the ceca where the bacterial counts were decreased by 2.4 log_10_ CFU/g. The bacterial reductions were also significant in ileum (1.36 log_10_ CFU/g on day 2) and colon (1.74 log_10_ CFU/g on day 3). Richards et al. (2019) were able to recover both phages of the cocktail for 5 days throughout the experiment in all three compartments of the chicken’s GIT, indicating successful self-replication *in vivo* and no competition between phages allowing them to co-exist. Overall, the time of phage application to limit *Campylobacter* colonization in chickens have been shown to be most effective over a 2- or 3-day period post phage administration (Loc Carrillo et al., 2005; El-Shibiny et al., 2009; Richards et al., 2019). In addition, when the time to slaughter after phage application was prolonged, numerous studies showed that cecal *Campylobacter* counts did not reach the counts detected in non-treated controls (Table 2). These findings suggest that phages’ success of self-replication is possible *in vivo* and can provide an antimicrobial safety net in cases when a slaughter day may be postponed. In addition, the diminished competitive advantage of the resistant types, as reported by Loc Carrillo et al. (2005), reinforces the hypothesis of Wagenaar et al. (2005) that the release of potent *Campylobacter* phages into the environment would not comprise any more significant risk.

Achieving complete elimination of *Campylobacter* in the bird GIT may be unrealistic with phage therapy for various reasons. However, the partial reduction could still be a productive outcome for reducing potential exposure to *Campylobacter*. This possible reduction can be estimated using quantitative microbial risk assessment (QMRA), which is the probability of infection and illness when a population (usually of humans) is exposed to pathogens in the environment (Chapman et al., 2016). QMRA is based on hazard identification, exposure assessment, dose-response, and risk characterization (Vose, 2008). Based on this approach, quantitative risk assessment models suggest that reducing two log_10_ CFU/g of *Campylobacter* in the ceca at the time of slaughter would significantly impact campylobacteriosis’s human incidence by approximately 30-fold (Rosenquist et al., 2003). Understanding how reductions in *Campylobacter* populations impact disease occurrence is essential, as, in nature, phages seldom eradicate their host bacterium populations (Connerton et al., 2008). The inability of complete microbial elimination may partly be due to natural bacterial resistance and the failure of phage particles to find host cells when occurring in low concentrations (Chibani-Chennoufi et al., 2004). Low concentrations of host cells should not be a concern when considering phage remediation in the ceca of broilers, as *Campylobacter* concentrations in the ceca often range from 4 to 8 log_10_ CFU/g (Rudi et al., 2004).

## Poultry Postharvest *Campylobacter* Phage Biocontrol

Postharvest application of lytic phages could selectively target *Campylobacter* populations without interfering with the remaining microbiota. Phage treatment can be used to inactivate *Campylobacter* attached to food contact surfaces or grown as biofilms. *Campylobacter* bacteriophages isolated from retail poultry have been used in some post slaughter experiments (Umaraw et al., 2017). While *Campylobacter* phages have been isolated from poultry carcasses, they occur in relatively low concentrations compared to the *Campylobacter* bacterial loads that have been reported on poultry skin in retail environments up to 10^4^ CFU per carcass (Dufrenne et al., 2001; Atterbury et al., 2003a; Scherer et al., 2006).

*Campylobacter* species can often be isolated from chicken skin and feathers, because the chicken skin has a protective effect on *Campylobacter* and other pathogens (Humphrey and Lanning, 1987; Berrang et al., 2000; Whyte et al., 2001; Atterbury et al., 2003a). This phenomenon is most likely due to the presence of feather follicles and skin folds that contain oils and fats that may protect bacterial cells from crystalizing during the freezing process. Since the infective dose of *Campylobacter* for humans is less than 500 cells, the research on the persistence of *Campylobacter* on chicken skin under freezing conditions is a significant food safety concern (Black et al., 1988). One of the approaches in the industry to decrease broiler carcass contamination includes the use of hyperchlorite in scald water and chillers (Atterbury et al., 2003a). However, this approach has been demonstrated to not significantly reduce pathogen loads, such as *Campylobacter* (Whyte et al., 2001). In addition, increasing the dilution of hyperchlorite increases its efficacy but reduces the quality of the product, which is intolerable by consumers (Atterbury et al., 2003a).

Host-specific phages have been successfully used in preharvest operations to control enteric *Campylobacter* counts in poultry (Table 2). Using a single phage therapy, Atterbury et al. (2003a) demonstrated over a one log_10_ CFU/cm^2^ reduction of *C. jejuni* on chicken skin inoculated with 10^6^ CFU of *C. jejuni* PT14 with the administration of 10^7^ PFU of group III phage φ2 when the skin was stored at 4°C for 10 days (Table 3). Atterbury et al. (2003a) reported an improved reduction to 2.5 log_10_ CFU/cm^2^ during additional cold storage (–20°C) of poultry skin, which was greater than the effect of cold storage without phage application. However, environmental conditions may have enhanced phage efficacy. It is commonly accepted that *Campylobacter* species cannot replicate at 4°C, and Atterbury et al. (2003a) findings verified that premise since the number of *C. jejuni* populations on chicken skin without phage stored at 4°C for 10 days were reduced by one log_10_ CFU. There was no increase in phage counts on any of the chicken skins inoculated with *Campylobacter*, indicating that phages may not reduce *Campylobacter* counts *in situ* without bacterial replication. The subsequent rationalization for the reduction in *Campylobacter* cells is that during inoculation, a fraction of phages effectively adsorbed to the surface of the host but did not replicate until the bacterium became more metabolically active. Their data supported this concept since no reduction was observed in either *Campylobacter* concentration or phage number when the phage was mixed with a non-susceptible bacterial host. The authors concluded that a combinatory phage cocktail of a broad host range must be used for the therapy to be practical. More so, combining phage application with freezing may cause further reduction of *Campylobacter* on broiler carcasses. In another study, Goode et al. (2003) demonstrated a more significant reduction of *C. jejuni* counts with a phage application than *C. jejuni* counts without phage due to the low temperature alone. Ninety-five percent reduction of *C*. *jejuni* occurred on chicken skin *via* group III phage 12673 at 10^6^ PFU/cm^2^ inoculated with 10^4^ CFU/cm^2^ *C. jejuni* C222 and incubated for 24 h compared to a non-phage treated group that resulted in a 90% reduction of *C. jejuni*, which was statistically different. Similar to the results of Atterbury et al. (2003a), the phages were able to persist on chicken skin over 48 h at 4°C. These results indicate that phage administration may lessen cross-contamination with pathogens from other carcasses and processing environments.

**TABLE 3:** *Campylobacter* phage treatments during the postharvest studies.

Phage	Group	Source	Administration	*Campylobacter* inoculation	Results	Resistance	Study
φ2	Group III	NCTC 12674, ACTC 35922-B2	10^7^ PFU/cm^2^	10^6^ CFU/cm^2^ *C. jejuni* PT14 on chicken skin	4°C >1 log_10_ CFU reduction 30 min, 3 days, 5 days	None	Atterbury et al., 2003a
					–20°C 2.3 log_10_ CFU reduction 5 days post administration		
NCTC 12673	Group III	NCTC	10^6^ PFU/cm^2^ on chicken skin	10^4^ CFU/cm^2^ *C. jejuni* C222 on chicken skin	4°C non-phage 90% reduction 24 h post administration	n/a	Goode et al., 2003
					4°C with phage 95% reduction 24 h post administration		
Cj6	Group not specified	Chicken feces	Low MOI (10) or	<100 cm^–2^ or	5°C(high MOI, high host density) >2 log cm^–2^ reduction 24 h post administration for cooked meat and 1.5 log cm^–2^ for raw beef	n/a	Bigwood et al., 2008
			high MOI (10^4^)	10^4^ cm^–2^ of *C. jejuni* FGCSCFT onto cooked or raw beef			
Cj6	Group not specified	Chicken feces	10^2^-10^8^ PFU mL^–1^	10-10^4^ CFU mL^–1^ of *C. jejuni* into tube with inoculum	24°C and 2 h post administration:	n/a	Bigwood et al., 2009
					1.8 × 10^5^ PFUmL^–1^ 3–8% reduction		
					1.2 × 10^6^ PFUmL^–1^ 33–52.3% reduction		
					1.1 × 10^7^ PFUmL^–1^ >96% reduction		
					Without phages 8.9% reduction		
CP8 or CP 30	Group III	Poultry excreta	10^6^ or 10^9^ PFU/ml	*C. jejuni* NCTC 11168 or PT14 at 10^5^ CFU/ml incubated at 37°C for 5 days grown in biofilms on glass	In biofilms: CP30 or CP8 versus 11168 or PT14	NCTC 11168 à CP8 (84%) à CP30 (90%) None in PT14	Siringan et al., 2011
					3 log_10_ CFU/cm^2^ reduction 2 h post administration		
					CP8 versus 11168 barely detectable limits 24 h post administration		
					CP8 versus PT14 1 log_10_ CFU/cm^2^ reduction 24 h post administration		
					CP30 versus PT14 2.5 log_10_ CFU/cm^2^ reduction 4 h post administration		
					Planktonic cells:		
					CP30 versus PT14 < 1 log CFU/cm^2^ reduction 4 h post administration		
F356 + F357	Group not specified	Free range poultry farms	10^7^ PFU/cm^2^	10^4^ CFU/cm^2^ of *C. jejuni* NCTC 12662 on chicken skin	0.73 log_10_ reduction at 5°C 24 h post administration	n/a	Zampara et al., 2017

Improvement of efficacy with a decrease in temperature was also noted in other meat matrices. For example, the use of phage Cj6 on raw beef at 5°C with high MOI and high host density reduced *C. jejuni* by 2.4 log_10_ CFU/cm^2^ in cooked meat and a 1.5 log_10_ CFU/cm^2^ in raw beef (Bigwood et al., 2008; Table 3). Conversely, at a high host density and high MOI, 2.8 CFU/cm^2^ at 6 h post phage administration and 2.2 log_10_ CFU/cm^2^ reductions at 24 h were observed on cooked and raw meat, respectively (Bigwood et al., 2008). However, at 24°C and a low host density, no significant reductions were observed even with a high MOI (Bigwood et al., 2008). The results were different from the *Salmonella* outcome in the same study where the introduction of *Salmonella* phage P7 produced a 4.7 log_10_ CFU *Salmonella* reduction when incubated at 24°C with a high MOI and low host density in cooked meat and two log_10_ reduction in raw beef. The decrease of *Salmonella* populations in the presence of the phage demonstrated that at low host cell load and high phage MOI, the number of bacterial cells eliminated does not rely upon the host cell load. These findings were consistent with Bigwood et al. (2009). This study also noted that inactivation of *Campylobacter* by phages continued and increased with time for 8 days incubated at 5°C for both cooked and raw types of meat. This finding is essential, as ready-to-eat meats are usually consumed within 7 days of purchase (Gilbert et al., 2007). In further work, Bigwood et al. (2009) concentrated on applying phage in the liquid food stored at room temperature for 2 h, as it is considered the maximum time for food storage at room temperature (Table 3). At the lowest phage concentration, the number of surviving host bacteria was close to 100%, but with the increasing concentration of phages, the inactivation of *C. jejuni* and *Salmonella* cells increased (Table 3). Decreased survival over time was observed for *C. jejuni* populations when the host concentration was low, indicating that with a given concentration of phages, the reduction of *Campylobacter* cells was more significant for lower concentrations of host bacterial cells.

In a more recent study, Zampara et al. (2017) demonstrated the application of lytic phages that targeted both CPS (group III) and flagella (group II) of *C. jejuni* on chicken skin under conditions that imitated a storage environment (Table 3). A combination of group III phages (F356 and F357) produced a 0.73 log_10_ reduction of *C. jejuni* counts on chicken skin at 5°C in 24 h. These results are in contrast to the results from CPS targeting phages used alone that produced a 0.55 log_10_ reduction (F356) and 0.49 log_10_ reduction (F357), and phage targeting the flagella (F379) that failed to reduce *Campylobacter* at a low temperature significantly. Previous studies have shown that motility may affect the infection proficiency of flagellotropic phages (Sørensen et al., 2015). Thus, the group II phage could be unsuccessful due to the potentially compromised motility of *C. jejuni* at lower temperatures. However, Zampara et al. (2017) observed that motility of *C. jejuni* was not a factor and further indicated that the temperature did not affect phage binding as no differences of phage binding between 37 and 5°C were observed within an hour of incubation. However, group III phages exhibited greater binding capacity with an average of 96% of phages adsorbing to the bacterial cells at 5°C after 24 h of incubation, compared to 55% of attached group II phages. The authors also observed an increase in free group II phage concentration after 24 h of incubation compared to 1 h incubation. They postulated that group II phages bound to the bacterial cell within the first hour but not permanently, explaining the increased concentration of free phages after 24 h.

Most phage studies involve the *Myoviridae* family of *Campylobacter* phages. However, Huang et al. (2020) characterized and described a rare member of the *Siphoviridae* family, CAM-P21, isolated from the beef grind. CAM-P21 was described to possess a broad host range, a better titer, and enhanced performance under diverse stress conditions compared to the *Myoviridae* family of phages. CAM-P21 reduced viable *C. coli* counts by more than two logs after a 12–24 h incubation period at both 42 and 37°C *in vitro*, respectively (Huang et al., 2020). The findings reported by Huang et al. (2020) suggest that a prospective combination of families of phages in a cocktail can potentially control for multiple *Campylobacter* species.

A significant concern for processing facilities is the buildup of biofilms on processing surfaces and equipment (Arnold and Silvers, 2000). *Campylobacter*, along with other pathogens, can form biofilms by producing a polysaccharide matrix (Gunther and Chen, 2009). In comparison to chemical sanitizers such as chlorine and peracetic acid, which appeared to be inefficient in removing biofilms, several bacteriophages have successfully reduced pathogen populations within biofilms (Deborde and Von Gunten, 2008; Siringan et al., 2011; Van der Veen and Abee, 2011). For example, bacteriophages CP8 and CP30 effectively reduced *C. jejuni* by one to three log_10_ in biofilms formed on glass surfaces (Siringan et al., 2011). While glass may not represent the surfaces typically found in poultry processing facilities, Siringan et al. (2011) detected a three log_10_ CFU/cm^2^ reduction within 2 h post phage administration in *Campylobacter* counts in biofilm incubated at 37°C using group II phages CP8 or CP30 (Table 3). However, bacterial cells were recovered at 4–8 h post phage administration that may be correlated with the reattachment of *C. jejuni* biofilm that was previously separated by phage treatment. Regardless of the final level of recovered *Campylobacter* cells after 4 h, instantaneous effects of phage application could still be used to initiate the dispersal of biofilms in poultry processing that high-pressure water treatments could follow. Remarkably, application of CP8 on *C. jejuni* NCTC 11168 resulted in nearly undetectable counts 24-h post phage application compared to *C. jejuni* PT14 strain, which produced a greater quantity of biofilm and exhibited less than one log_10_ CFU/cm^2^ reduction over 24 h period in 11168 counts when CP8 phage was used. However, CP30 reduced *C. jejuni* PT14 counts by 2.5 log_10_ CFU/cm^2^ in 4 h regardless of the excessive amount of biofilm matrix.

Interestingly, although CP30 showed such success on *C. jejuni* PT14 cells in biofilm, the phage produced less than one log_10_ CFU/cm^2^ reduction in the planktonic cells under the same conditions. This finding contradicts previous observations by Sharma et al. (2005) that concluded similar effects of phages on attached and planktonic cells. Furthermore, similarly to several preharvest studies, there appeared to be stasis in phage concentration in biofilm and planktonic cultures throughout the experiment regardless of the reduced *Campylobacter* numbers. This phenomenon indicates a threshold above which phage counts do not increase despite being constantly supplemented into the matrix through replication. This could illustrate a passive biocontrol in which the quantity of phages is adequate to decrease cell counts without the necessity for excessive levels of phage replication.

Biofilms represent an accumulation of various cells enclosed by a matrix of extracellular polymeric substance (EPS) produced by bacterial members of the biofilm (Sillankorva et al., 2011). The main constituents of the EPS are long-chain sugars, DNA, and other various biological macromolecules that can be very diverse (Flemming, 2008). Bacteria within a biofilm have demonstrated high resistance to antibiotics and other antimicrobial agents (Harper et al., 2014). In addition, the concentration of the respective agent required to generate antimicrobial effects can be more than a thousand times higher than the amount necessary for free-living microorganisms (Ceri et al., 1999). Yet, bacteriophage application has shown high success in biofilm dispersal within bacterial species (Siringan et al., 2011).

Since biofilms’ extracellular contents depend on the microbial populations present, phage cocktails should be evaluated against biofilms comprised of various bacterial genera. The success of specific phages in removing biofilms can be potentially due to polysaccharide depolymerase production, which breaks up the polysaccharide matrix (Hughes et al., 1998). For instance, many caudovirales, such as T4 and HK620 of *E. coli*, possess a polysaccharide depolymerase protein at the end of their tail that can degrade microbial capsules and allow cellular attachment (Harper et al., 2014). Lu and Collins (2007) engineered such a phage that stimulated host polysaccharide depolymerase expression resulting in the breakup of the polysaccharide matrix. They also reported that its application reduced *E. coli* biofilms by nearly 100% and produced a reduction two times better than non-enzymatic phage (Lu and Collins, 2007). The polysaccharide depolymerase expressing phages may improve efficacy in preharvest strategies where the sustained phage replication is less expected, such as the poultry GIT. However, host specificity insinuates that a thorough library of phages must be preserved so that a proper administration can be designated for each bacterial community within a biofilm. The possibility of combinations within biofilms suggests that it may be challenging for any created phage to be successful on a wide range of biofilms; although, the concept could work with other biofilm-destroying enzymes (Lu and Collins, 2007). Future developments for the strategy may incorporate several phage promoters amplifying enzyme production that would target multiple EPS constituents and target multiple bacterial species (Lu and Collins, 2007). Unlike T7 *E. coli* phage, *Campylobacter* phages do not possess RNA polymerase and there are no strong promoters that have been identified within the *Campylobacter* phages, which would make genome cloning within the phage difficult.

## Potential *Campylobacter* Phage Host Resistance Mechanisms

A potential concern of phage resistance arises from the increased or prolonged phage application for medical applications or in the food industry (Goodridge and Bisha, 2011). In the environment, bacteria and bacteriophages exist in a co-evolution cycle, in which phage-insensitive hosts survive or prevent phage predation by passing on the corresponding resistance mechanisms (Weinbauer, 2004; Labrie et al., 2010). Phage resistance usually arises due to the loss or modification of cell surface molecules, such as capsules, LPS, pili, or flagella (Labrie et al., 2010).

Labrie et al. (2010), Bradde et al. (2017), and Jiang and Doudna (2017) briefly described general phage resistance mechanisms. The mechanisms can be classified into two categories: (1) prevent initial phage interaction with the host and/or (2) survive phage infection. The former is commonly accomplished *via* modification of phage receptor sites, preventing DNA entry by changing the injection site conformation, and producing physical barriers through the extracellular matrix (Labrie et al., 2010). For instance, the MeO*P*N moiety of the CPS has been identified as a receptor site for lytic phages, such as F336 (Sørensen et al., 2011, 2012). Sørensen et al. (2012) observed phage resistance when the phase variable phosphoramidate (MeO*P*N) moiety of CPS of *C. jejuni* was modified. In addition, over 70% of *C. jejuni* were found to possess modifications in their MeO*P*N moiety (McNally et al., 2007; Alphen et al., 2014). Although these surface structures often function as virulence factors and can contribute to bacterial survival, studies have shown that phage resistant *C. jejuni* have a competitive disadvantage in terms of fitness compared to phage sensitive isolates in the same environment without the phage’s presence (Levin and Bull, 2004; Scott et al., 2007a,b; Carvalho et al., 2012; Hooton et al., 2020).

Scott et al. (2007b) showed that phage therapy affects the growth of resident *Campylobacter* in the avian GIT. Scott et al. (2007b) showed that amongst *C. jejuni* that survive phage infection in broiler chickens are phage-resistant types that exhibit genomic rearrangements. Scott et al. (2007b) investigated group III phage CP34 predation on *C. jejuni* HPC5 and R14-CampMu and R20-CampMu on R14 and R20 *C. jejuni* strains within the avian GIT and isolated phage-resistant mutants. These mutants were most likely not the dominant *Campylobacter* strain; since the mutations primarily altered the flagella, which caused a significant negative impact on colonization. The authors concluded that phage resistance is rare in poultry because the mutants that avoid phages are not capable of chicken GIT colonization and quickly mutate back to colonization-capable sensitive forms.

Prevention of phage infection is not the only bacterial response utilized. Other phage resistance mechanisms focus not on preventing phage entry but instead on bacterial host survival once infected by phage. Clustered regularly interspaced short palindromic repeats (CRISPR) have been studied extensively and provide a mechanism for bacteria to survive multiple phage infections (Barrangou et al., 2007). CRISPR loci are present in 45% of the bacteria, according to Grissa et al. (2007). Initially observed in *E. coli* and described by Ishino et al. (1987), CRISPR is an arrangement of short repeated sequences split by spacers with unique sequences and are located in plasmid and chromosomal DNA. These spacers are frequently the nucleic acids of plasmids and viruses (Rath et al., 2015). CRISPR activity involves the CRISPR-associated (*cas*) genes located adjacent to the CRISPR that code for proteins fundamental for the proper immune response (Barrangou et al., 2007). There are distinct types of CRISPR systems. *C. jejuni* NCTC 11168 and PT14 have been reported to contain subtype II-C CRISPR systems that lack Cas4 proteins (Dugar et al., 2013). While the absence of Cas4 has been noted in subtype II-C CRISPR systems, the protein has been shown to possess an exonuclease activity which is required for CRISPR adaptation (Shah et al., 2013). Thus, the deficiency of Cas4 may inhibit spacer integration, such as phage defense.

By studying carrier state life cycle (CSLC) of *C. jejuni* PT14 for CP8 and CP30A phages, Hooton and Connerton (2015) had the opportunity to assess the process of the CRISPR-Cas system in the company of Class III phage carrying Cas4. The CSLC represented a mixture of bacteria and phages in a state of equilibrium (Lwoff, 1953). Under CSLC conditions, phages could continue to associate with a well-suited host and generate free virions in a search for new hosts (Siringan et al., 2014). While a fraction of the bacteria attain resistance, some sensitive cells happen to maintain the phage population so that both survive (Siringan et al., 2014). Although appearing as lysogens, strains exhibiting CSLC do not integrate phage nucleic acid into the host genome (Siringan et al., 2014). In addition, CSLC has been strictly observed with the lytic phages, and there are several experimental examples of such relationships (Li et al., 1961; Jones et al., 1962; Jarling et al., 2004; Bastías et al., 2010). During their experiment, Hooton and Connerton (2015) observed that the carrier state populations was comprised of bacteria that had extended the CRISPR array by acquiring naive spacers. Markedly, all the new spacer sequences could be located in the host genome sequence and were not noted in either co-propagating phage genomes. This mechanism prevents phage DNA inclusion, which allows the phage to replicate undetected (Hooton and Connerton, 2015). The phenomenon indicates that even internal host resistance mechanisms can be complicated and mechanistically elusive to understand. The authors could not conclude how long bacteria carrying these spacers survived (Hooton and Connerton, 2015). Potentially, the CRISPR facilitated immunity is sustained in this setting as a self-sacrificing response to the constant exposure to phage infection but this still does not explain why the phage resides as a practical component of the system. Hooton and Connerton (2015) suggested that *Campylobacter* phages can use Cas4-like protein as an anti-CRISPR technique to initiate a spacer integration to use host DNA as an operational distraction to phage DNA. Therefore, *Campylobacter* that obtain self-spacers and avoid phage infection must overcome CRISPR-facilitated immunity against itself by either withstanding alterations in gene regulation or losing the interference functions exposing them to the foreign DNA invasion (Hooton and Connerton, 2015).

Other factors may be associated with phage resistance. It has been insinuated that the persistence of phage infection in microbial cultures could be facilitated by gradual adsorption rates of the phages, permitting the bacterial host time to replicate before the cellular machinery is overcome (Torsvik and Dundas, 1980). Remarkably, bacteriophages generated by *Campylobacter* CSLC strains retained phage adsorption constants comparable to those propagated by traditional lysis (Siringan et al., 2014). Siringan et al. (2014) showed that 70–90% of the phage population in *Campylobacter* CSLC strains were closely related with their host, either bound to CPS of the cell or preserved within the host cell with the potential that the phage genomes are carried as episomes (Siringan et al., 2014). Siringan et al. (2014) proposed that disbanded phage elements are associated with the bacteria, and the outcome of the phages is not subject to the fate of the host. Nevertheless, the authors also suggested that the presence of the phages is not totally passive, and that the host’s replication contributed to the detected phage concentration. Phenotypic analysis of the CSLC *Campylobacter* strains showed that the cells were non-motile, and their flagella were shortened (Siringan et al., 2014). These findings are consistent with impaired motility of most phage-resistant types retrieved from post-infection cultures, where mutants with non-functional flagella have been shown not to establish phage infection (Coward et al., 2006; Scott et al., 2007a,b). Consequently, CSLC strains were incapable of colonizing chickens in the Siringan et al. (2014) study. These features make CSLC strains an essential ecological reservoir for phage propagation and conceivable commercial interest as a constant source of phages for remedial and biological sanitation purposes in the food and farming productions directed at reducing human exposure *to Campylobacter*.

An interesting observation was noted by Burmeister et al. (2020), indicating an evolutionary compromise between phage and antibiotic resistance in bacteria. When studying the interactions between phage and antibiotic resistance genes, Burmeister et al. (2020) observed increased antibiotic sensitivity in bacteria where the phage resistance emerged. The relationships between phages, antibiotic-resistant bacteria, and antibiotic-sensitive bacteria are complicated. Occasionally when phage resistance develops, the bacteria increase antibiotic sensitivity (Burmeister et al., 2020). To achieve a better understanding of these associations, Burmeister et al. (2020) screened 33 commercial and environmental *E. coli* phages for their dependence on the antibiotic efflux pump gene *tolC*. They identified phage U136, which depends on the core of the LPS and the antibiotic resistance gene *tolC*. Burmeister et al. (2020) also noted that U136B selects host mutants with genes encoding its essential host entry elements, *tolC* and LPS. These phage-resistant mutants exhibited phenotypic modifications to their tetracycline sensitivity, which was facilitated by *tolC*, as well as colistin, stimulated by LPS associated elements, or both (Burmeister et al., 2020). These results demonstrate the potential to reverse the existing antibiotic resistance and potentially alleviate some of the public health problems associated with treatment by antibiotics.

Lysogenic bacteriophages of *Campylobacter* species have been the emphasis of numerous studies directed at comprehending the complicated relations that have become established between bacteria and viruses throughout the millions of years of co-existence (Hooton et al., 2020). Studying lysogenic phages of *Campylobacter* species have been used to assess different protein expression responses in host cells, genomic reshuffling, and methods of resistance to phage infection in *Campylobacter* species such as CRISPR-facilitated immunity and phase variation (Hooton et al., 2020). *C. jejuni* integrative elements (CJIE) and Mu-like phage sequences have been previously detected in *Campylobacter* species (Parker et al., 2006; Scott et al., 2007b; Clark et al., 2012). Genomic rearrangements triggered by Mu-like prophages are considered as main features for administering host resistance to phage infection (Hooton et al., 2020). It is commonly recognized that integrated phages can potentially change the virulence phenotype of the host (Brussow et al., 2004). For example, a study implicating *C. jejuni* encoding homologs of Mu-like phages (CJIE1) indicated increased adherence and invasion of cells compared to *C. jejuni* cells lacking integrated phage components (Clark et al., 2012). However, in an analogous study, no statistical differences were detected between the adhesion and presence of CJIE1-like elements (Skarp et al., 2017). In addition, genomic rearrangements by CJIE1-like prophages contributed to the host resistance to phage infection in *Campylobacter* and have been observed to hinder the host’s ability to attain extracellular DNA through the natural transformation process (Scott et al., 2007b; Gaasbeek et al., 2009, 2010; Brown et al., 2015).

*Campylobacter* use various strategies to avoid phages, such as genetic rearrangements, utilization of alternate flagellin, phase variation, and attainment of CRISPR spacers to abolish phage predation (Scott et al., 2007b; Hooton and Connerton, 2015; Lis and Connerton, 2016; Gencay et al., 2018). More recently, researchers from Singapore-MIT Alliance for Research and Technology (SMART) discovered another new and remarkable type of bacterial defense system. The SspABCD-SspE phosphorothioate (PT) system is different from previously studied mechanisms. For instance, SspE protein inhibits phage replication by nicking the phage DNA rather than degrading dsDNA as seen in previous mechanisms and the protection against bacteriophages is stimulated by sequence-specific PTs (Xiong et al., 2020). Xiong et al. (2020) described this unique defense system for *Vibrio cyclitrophicus*, *E. coli*, and *Streptomyces yokosukanensis*, which have different genetic structures, metabolism, and phenotypes. These findings have expanded the understanding of the diversity of a bacterial defense system. In summary, when designing a phage therapeutic cocktail, the discovered bacterial defense mechanisms must be considered.

## *Campylobacter* Phage Resistance in Poultry Production and Strategies for Circumvention

The use of phages to control *Campylobacter* in poultry deviates from the more clinical applications of phage therapy because the bacteria are not explicitly pathogenic in birds they reside as a part of the GIT. *Campylobacter* colonize the chicken intestine to a high density and are certainly an optimal phage therapy target (Connerton et al., 2011). However, concerns have been raised that *Campylobacter* will merely develop resistance to phages, making this strategy ultimately ineffective in the long term (Barrow, 2001). Although a multiplicity of spontaneous phage-resistant bacterial mutants develops in *in vitro* populations, resistance to phages has been associated with reduced virulence *in vivo* and reduced survival (Adams, 1959; Connerton et al., 2004; Loc Carrillo et al., 2005; Capparelli et al., 2010). Unlike bacterial resistance to bacteriostatic chemical agents, phages continually evolve to evade host barriers, leading to an evolutionary balance that allows both host and phage to multiply. To achieve success in phage therapy, modifying the balance in favor of phage serves as a momentary opportunity to reduce bacterial numbers, at least in the short term. For the application in preharvest procedures, the return to equilibrium can potentially be avoided by the slaughter of the birds while the *Campylobacter* populations are still reduced and have not yet recovered.

In practice, the required replication for successful phage treatment has its advantages and disadvantages. The benefits are that phage application is economical as there is no need to match the administration dose to the concentration of *Campylobacter* colonization in the bird because the phages will propagate according to the number of hosts present (El-Shibiny et al., 2009). A potential disadvantage is the possible development of bacterial host resistance due to the increased number of replications (El-Shibiny et al., 2009). However, El-Shibiny et al. (2009) stated that this specific disadvantage did not appear to be a concern even 5 days post phage administration during their experiment. Furthermore, the authors hypothesized that the slaughter of birds 2 days following phage therapy would be the most optimal management approach by allowing phage replication and decrease the risk of developing bacterial resistance. Other *in vivo* preharvest studies support this approach (Table 2). Thus, phage application closer to the end of poultry production can mitigate selective pressure on *Campylobacter* and prevent the transmission of infection to other birds.

Another strategy to circumvent the potential problem of developing phage resistance is administering phages from different phage groups. Thus, group II and group III phages are capable of binding multiple host cell receptors (Coward et al., 2006; Sørensen et al., 2011). Hammerl et al. (2014) showed that the application of a phage cocktail consisting of the same group of phages contributed to a higher resistance frequency versus the application of one type of phage and a combination of different groups of phages (Table 2). However, the administration of group III phage alone or in combination with group II phage yielded a lower resistance rate than when applied in combination with another group III phage (Hammerl et al., 2014). In addition, Hammerl et al. (2014) observed that the resistance to group II phages was much more constant than resistance to phages of group III, which was similar to reports from other studies (Loc Carrillo et al., 2005; Fischer et al., 2013), where host cells rapidly reverted to susceptible types. Therefore, it would be beneficial to examine the resistance potential of all phages of a cocktail to construct the final cocktail of phages from different groups, which vary in their host ranges, lytic spectra, and resistance mechanisms. Furthermore, postharvest phage application to poultry meat may restrict the emergence of phage-resistant strains since *C. jejuni* cannot grow at lower temperatures (Atterbury et al., 2003a). Thus, phage-resistant variants of *C. jejuni* cannot arise under these conditions (Atterbury et al., 2003a).

Several pathogens, such as *C. jejuni* have a phase variation (PV) mechanism that allows the bacterium to rapidly adapt to external environment, specifically for a host-associated bacteria, through modifications of surface structures (Sandhu et al., 2021; Sorensen et al., 2021; Yamamoto et al., 2021). PV occurs *via* hypermutation of simple sequence repeats (SSR) through slipped-strand mispairing during DNA replication (Sandhu et al., 2021). The PV mechanism involves differences of protein expression in an on-off fashion and is located within protein-coding regions within genes that regulate the expression of surface structures, such as CPS and flagella (Parkhill et al., 2000). PV can affect phage infection, as surface structures function as receptors for phages. Due to PV, treatment of birds with phages have shown to produce high degree of resistance by modifications in the formation or expression of receptors (Sandhu et al., 2021).

Through computational analysis considering different mutational structures of the PV within *C. jejuni* and phage F336 interaction in a nutrient-controlled continuous culture system, Sandhu et al. (2021) studied how phage infections effect the evolution of PV in *C. jejuni*. The authors observed that extremely low and extremely high mutation rates are evolutionarily unfavorable and rather evolutionary stable mutation rates are affected by fluctuating density of the phage and the reduction of bacterial numbers. The equilibrium between counter-selection and phage infection can result in the progression of PV phage receptor and maintenance of the PV receptor-dependent phage (Sandhu et al., 2021). From a practical standpoint, Sandhu et al. (2021) predicted that introducing phage into the system with no continuing bacterial growth, such as in post-harvest applications, may result in nearly 10-fold reduction in bacterial counts. Whereas pre-harvest application of F336 phage should be considered when counter-selection acts only on the phage resistant variants of *C. jejuni* strains or in combination with another phage that binds F336-resistant variants (Sandhu et al., 2021).

Interestingly, Sorensen et al. (2021) indicated that PV *Campylobacter*-dependent phages can imitate their hosts and avoid bacterial resistance. Sorensen et al. (2021) observed that the *Fletchervirus* genus from *Myovirideae* family of *Campylobacter* phages similar to their host contain hypermutable traits, which influence phase variable expression of some of the receptor-binding proteins. In addition, the resulting phenotypically varied phage populations contain sub-populations that can infect the host when PV-receptor is not present, supporting co-existence of phage and host in the shared environment (Sorensen et al., 2021). Phenotypic variability that can be generated by PV can limit duplicability of results with *C. jejuni*. Currently, there does not appear to be routine commercial applications for *Campylobacter* phages, due to *Campylobacter* phase variation and genomic instability. However, this may be resolved with the latest research from Yamamoto et al. (2021) demonstrating a potential to stabilize the *Campylobacter* genome in one phase variation state that could be used to devise practical phage applications.

## Future Strategies for Optimizing Phage Application in Poultry Production

*Campylobacter* phage biocontrol for poultry production appears to have promise. However, several strategies involving current and future developments need to be explored to optimize their efficacy from a practical standpoint. These current and future strategies represent some possible development strategies to improve phage effectiveness and potentially achieve routine commercial application in poultry. One of the considerations for practical phage therapy is developing the appropriate delivery system to apply the bacteriophages. Since phages are self-replicating, single dosing of *Campylobacter* phages in birds may be sufficient, though it depends on high bacterial concentrations (Loc Carrillo and Abedon, 2011). When and where to apply *Campylobacter* phages in poultry processing requires strategic approaches that consider the biology of the bacteriophage and its interaction with target host cells. Capparelli et al. (2010) found an inverse relationship between the incidence of lytic phages and their hosts. Different from what happens with antibiotics, administration of phages when the bacterial count is low may be ineffective. Thus, one phage dose may be appropriate only when the target bacterial population divides rapidly while multiple phage administration doses are employed when bacteria divide slowly (Capparelli et al., 2010). In contrast, Bigwood et al. (2009) observed that a certain threshold of phages was sufficient to decrease the *Campylobacter* counts without the need of excessive replication. In addition, the reduction of *Campylobacter* cells was more significant for lower concentrations of host bacterial cells in the liquid culture. Conveniently, because of their low toxicity and resistance to degradation, phages can be supplied within the drinking water or feed to allow for continuous dosing throughout the rearing period (Kittler et al., 2013).

More radical approaches for applying *Campylobacter* phages in poultry may offer promise to overcome some of the issues encountered with general phage therapy, such as the development of host cell resistance. For example, phage endolysins have a broader host specificity than lytic phages (Siringan et al., 2011). Along these lines, Zampara et al. (2021) suggested the application of phage-derived enzymes instead of lytic phages to overcome some of the problems inherent with intact phages. Endolysins are phage-programmed enzymes that destroy the peptidoglycan layer when externally added leading to osmotic imbalance and cell death (Zampara et al., 2021). Although endolysins have successfully been used in an antibacterial application and can exhibit low development of resistance, their application is currently more suitable toward Gram-positive bacteria, as Gram-negative bacteria contain an outer membrane that inhibits entry of endolysins to the peptidoglycan layer (Gutiérrez and Briers, 2021). Previous studies have successfully utilized fusion of endolysins with binding domains of bacteriocins or polycationic and amphipathic peptides to surmount the outer membrane barrier of Gram-negative bacteria (Lukacik et al., 2012; Yan et al., 2017; Heselpoth et al., 2019). Recently, Zampara et al. (2020) demonstrated successful application of the fusion of phage T5 receptor-binding protein (RBP) and endolysin (Innolysins) against *E. coli*. In their study, the bactericidal activity proved to be dependent on the phage T5 RBP cognate receptor, FhuA.

Based on Zampara et al. (2020) reporting of a successful bactericidal effect of Innolysins against *E. coli*, they (Zampara et al., 2021) applied the same concept against *C. jejuni*. Zampara et al. (2021) demonstrated that the H-fiber derived from a *C. jejuni* integrated elements (CJIEs)-1 like prophage CAMSA2147 functions as a unique RBP. Zampara et al. (2021) designed Innolysins that target *C. jejuni* by fusing the H-fiber and T5 endolysin. Zampara et al. (2021) noted that the application of Innolysins on chicken skin at 5°C contaminated with *C. jejuni* CAMSA2147 led to an average of 1.4 log_10_ reduction of bacteria, indicating that Innolysins can eradicate *C. jejuni in situ*. Zampara et al. (2021) concluded that the H-fiber potentially recognizes a different receptor on the host cells than the lytic *C. jejuni* phages that bind to the host cell CPS or flagella.

Phage intervention strategy may also be advantageous during the somewhat static steps of poultry processing, where slow *Campylobacter* growth occurs, such as poultry plant surfaces that may contain biofilms, at the endpoints of processing, and poultry plants that utilize reuse water systems. Phage application can be exceptionally favorable in water reuse systems, which use filters to remove large chemical particles but would allow phages to pass through, offering the potential for a single cocktail to be utilized multiple times as an ongoing component of the recycled water (Mannapperuma and Santos, 2004; Meneses et al., 2017; Micciche et al., 2018). Theoretically, phages can persist in water indefinitely, although inactivation does occur (Pinon and Vialette, 2018). Thus, the investigation of phage biocontrol’s reuse possibilities within poultry processing water, such as carcass wash waters and chilled water, is warranted. Optimizing *Campylobacter* phage administration in poultry processing water and reuse water will require determining whether inactivation problems occur and can be overcome. It also would be worth determining whether *Campylobacter* phage can remain active in the presence of antimicrobials such as acids typically used in poultry processing plants. If phages remain viable and sustain their lytic properties, the potential of introducing phages as a component of multiple hurdle interventions that include non-phage antimicrobials such as acids would be highly attractive.

Bacteriophages may hold other advantages for application in the poultry processing plant. Due to their very low toxicity and specific host range (Loc Carrillo and Abedon, 2011), large quantities of phages could be added to processing waters with little to no risk to workers, which can concern when employing traditional chemical sanitizers. Most lytic phages, including *Campylobacter* phages, do not produce toxic by-products and have little to no harmful effect on humans or birds (Skurnik et al., 2007; Abedon et al., 2011; Loc Carrillo and Abedon, 2011). However, it has been noted that phages can interact with host immune systems, resulting in a harmful but reversible immune response (Alisky et al., 1998; Kutateladze and Adamia, 2010). In addition, since phages are grown and isolated from cultures containing pathogenic host cell bacteria, improper purification can lead to pathogenic bacterial components triggering severe immune responses (Skurnik and Strauch, 2006; Skurnik et al., 2007). To overcome this, ion-exchange chromatography or high-speed centrifugation can ensure separation between the phage and residual bacterial components (Bogovazova et al., 1992; Sulakvelidze et al., 2001). Regardless, in preharvest environments, appropriate controls will need to be implemented to optimize the application of phages and the immune response. In addition, unlike traditional sanitizers and regardless of concentration, phages have no deleterious impacts regarding food quality (Greer, 1986, 2005; Barrow and Soothill, 1997).

## Conclusion

Quantitative risk assessments have concluded that reductions in *Campylobacter* both pre-and postharvest poultry production would potentially mitigate the health risk posed by campylobacteriosis (Havelaar et al., 2007; Lake et al., 2007; Nauta and Havelaar, 2008; Nauta et al., 2009). While bacteriophages may not eliminate *Campylobacter* from chicken ceca or carcass, their ability to reduce bacterial counts represents a promising avenue for eliminating the risk of contamination from a finished product. The precise mechanisms of *Campylobacter* phage lytic activity and host cell resistance must be elucidated to utilize phages for widespread control of *Campylobacter*. Some of these may be overcome by a targeted selection of *Campylobacter* phages and phage cocktails that contain multiple *Campylobacter* families or groups of phages with a wide range of specificities to accommodate host cell variability. Development of non-phage technologies such as isolation of endolysins and fusion with various host cell binding proteins may offer a novel strategy that overcomes *Campylobacter* host cell resistance in a more general fashion.

Evaluation of phage cocktails should also be investigated to remediate biofilms on processing surfaces. However, biofilm extracellular composition depends on the microbial populations present. Therefore, phages that code for polysaccharide depolymerase may effectively be applied with other phages against biofilms comprised of diverse microbial populations. As such, *Campylobacter* phage cocktails should be evaluated against biofilms that contain multiple bacterial genera to assess if *Campylobacter* population reductions are still observed in the presence of these mixed bacterial populations. Ultimately, mixtures of *Campylobacter* phages and non-*Campylobacter* phages may need to be employed to overcome biofilms that consist of fairly complex microbial ecosystems. It would be interesting to determine whether synergism for disruption of these more complex biofilms would occur when a multiple mixed phage cocktail is applied.

*Campylobacter* phage treatment can be implemented in poultry live bird production and processing operations with appropriate safety and quality practices. Nevertheless, understanding how phages acquire resistance and specifically how *Campylobacter* phages infect their host is essential for optimizing the efficacy under commercial conditions. As *Campylobacter* phage biology is better understood, optimizing their routine application in poultry production should be achievable. Indeed, better search tools for isolating *Campylobacter* phage with more broad-spectrum host specificity and optimizing delivery systems for maximum efficacy should offer incremental improvements. However, equally important will be the strategic application of *Campylobacter* phages within the various phases of poultry production. For example, postharvest employment during poultry processing may result in reducing risk more effectively than elsewhere. However, even within poultry processing, certain stages such as those that involve carcass rinses or reuse water may be more optimal delivery systems. With potential developments in phage technologies and a more strategic application management approach in both pre-and postharvest poultry environments, *Campylobacter* phages offer a viable potential hurdle for administration at multiple places throughout poultry production.

## Author Contributions

EO wrote the latest version of the manuscript. SR helped with writing and editing of the manuscript. AM, YY, and MR contributed to the first draft of the manuscript. All authors listed approved the manuscript for publication.

## Conflict of Interest

The authors declare that the research was conducted in the absence of any commercial or financial relationships that could be construed as a potential conflict of interest.

## Publisher’s Note

All claims expressed in this article are solely those of the authors and do not necessarily represent those of their affiliated organizations, or those of the publisher, the editors and the reviewers. Any product that may be evaluated in this article, or claim that may be made by its manufacturer, is not guaranteed or endorsed by the publisher.
